# Lung adenocarcinoma patients with KEAP1 mutation harboring low immune cell infiltration and low activity of immune environment

**DOI:** 10.1111/1759-7714.14089

**Published:** 2021-07-30

**Authors:** Wanwan Cheng, Bin Xu, Haitao Zhang, Shencun Fang

**Affiliations:** ^1^ Department of Respiratory Medicine, Nanjing Chest Hospital The Affiliated Brain Hospital of Nanjing Medical University 215 Guangzhou Road Nanjing Jiangsu 210029 China

**Keywords:** immunotherapy, Kelch‐like ECH‐associated protein 1, lung adenocarcinomas, tumor microenvironment

## Abstract

**Background:**

Kelch‐like ECH‐associated protein 1 (KEAP1) has been identified as a cancer driver gene in lung adenocarcinoma (LUAD), and increased evidence has given us clues about the association of KEAP1 mutation and immune characteristics. We assessed the association between KEAP1 mutation and tumor microenvironment in LUAD systematically.

**Methods:**

With the data collected from The Cancer Genome Atlas (TCGA), we evaluated the association of KEAP1 mutation with tumor infiltrating leukocytes (TILs), including dendritic cell, CD8 T cell, CD4 T cell, neutrophil, B cells, and macrophage. Expression differences of the markers of those immune cells were also measured. We further compared the expression of antigen presentation genes and chemokines and the enrichment score of immune‐related pathways.

**Results:**

KEAP1 mutation had significant association with lower TILs and cytotoxic T lymphocyte. Strikingly, almost all of antigen presentation genes and chemokine showed lower expression in KEAP1‐mutated tumors. Moreover, most of immune‐related pathways were less active in KEAP1‐mutated tumors. As expected, KEAP1‐wild type LUADs favored better overall survival after immunotherapy. Finally, one patient harboring KEAP1 mutation along with a lack of immune cells infiltration in tumor microenvironment failed to respond to checkpoint inhibitor despite high tumor mutational burden (TMB).

**Conclusions:**

KEAP1 mutation has a significant effect on the tumor immune milieu of LUAD and may play as a predictive biomarker of immunotherapy for LUAD patients.

## Graphical abstract

Immune cells, the key markers of immune cells, and HLA molecules show significantly down‐expressed in KEAP1‐mutated LUADs. Not only the multiple immune‐related biological processes, but also the interaction between chemokines and their receptors have shown KEAP1 mutation could impair the immune pathways. KEAP1‐wild type LUADs favored better overall survival after immunotherapy.

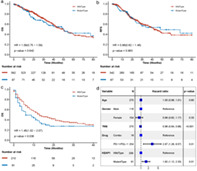

## INTRODUCTION

Lung cancer is a leading cause of cancer‐related mortality in the world, with ~2 093 000 newly diagnosed cases in 2018.^1^ Non‐small cell lung cancer (NSCLC) accounts for 80%–85% of lung cancer with a poor 5‐year survival rate of ~18%.^2^ Among NSCLC, the incidence rate of one of its major histological subtypes, lung adenocarcinomas (LUAD), is ~50%. Recent advances in next generation sequencing (NGS) have revealed many vulnerable gene alternations in LUAD including the targetable ones such as the epidermal growth factor receptor (EGFR) gene and translocations of the anaplastic lymphoma kinase (ALK) gene.^3^ Apart from them, the alternation of Kelch‐like ECH‐associated protein 1 (KEAP1) gene, encoding the KEAP1, has been found to be associated with LUAD with a frequency of 23% and therefore is considered a cancer driver gene.^4^ Under normal conditions, it is identified as a substrate adaptor for a Cul3‐containing E3 ubiquitin ligase and its main function is to complex with nuclear factor erythroid‐2‐related factor 2(Nrf2) through a direct protein–protein interaction, maintaining the latter at a low level.^5^ Because Nrf2 is responsible for organizing a wide range of cellular defense processes, the Nrf2–Keap1 pathway has been considered a major cellular defense pathway.

In recent years, new treatment modalities in NSCLC, such as immunotherapy or its combinations with others like chemotherapy, have strikingly induced durable responses.^6^ Specifically, treatments based on immune checkpoint blockade have demonstrated high efficacy in form of a variety of inhibitors such as ipilimumab (anti‐CTLA4), nivolumab (anti‐PD1), pembrolizumab (anti‐PD1), atezolizumab (anti‐PD‐L1), and avelumab (anti‐PD‐L1). Likely because of the heterogeneities of inter‐ and intra‐tumors as well as varied tumor micro‐environment (TME), only a specific subset of patients can benefit from such treatments, unfortunately. As a result, intensive research has been done to identify potential biomarkers for selecting the patients who are expected to respond well to such treatment modalities. Several studies have shown that PD‐L1 expression assessed by immunohistochemistry (IHC), tumor mutational burden (TMB) by NGS, microsatellite instability (MSI) by PCR or NGS, as well as deficient mismatch repair (dMMR) by IHC were associated with response to cancer immune checkpoint blockade treatment.^7^, ^8^, ^9^ Specifically, PD‐L1 expression and TMB are being intensively investigated in NSCLCs. In addition to these two biomarkers, enthusiasm in elucidating the components in TME, such as tumor‐infiltrating lymphocytes (TILs) and specific immune genes and pathways, has been reinvigorated, and potential biomarkers are being explored for the identification of patients that benefit from immune checkpoint inhibitors (ICIs) and therapies targeting angiogenesis. Although, as a commonly altered gene like TP53, KRAS, and EGFR in LUAD,^10^ changes within tumor immune milieu, and accordingly the clinical implication relevant to the mutation of KEAP1, remains largely unknown. Specifically, the studies are limited to investigating the association between mutations of some specific genes like KEAP1 with TME and the clinical benefit and potential drug targets.

To understand the effect of KEAP1 mutation on the immunity of LUAD, we have associated TIL score with KEAP1 alternation; furthermore, relevant immune marker, human leukocyte antigen (HLA), immune‐related pathway and cytokine networks. Finally, Cox models have been applied to assess associations between KEAP1 mutation and disease prognosis and the prediction of patient survival after immunotherapy in LUADs.

## METHODS

### Data sources

The data for somatic mutation, RNA sequencing (RNA‐seq) expression and clinical information was downloaded from the University of California Santa Cruz (UCSC) Xena (https://xenabrowser.net/datapages/). The somatic mutations were filtered to keep only those variant types including frameshift deletion, frameshift insertion, in‐frame deletion, in‐frame insertion, missense mutation, nonsense mutation, nonstop mutation, splice site, translation, and start site. A cohort of patients who received immunotherapy was collected from cBioportal (https://www.cbioportal.org/).^11^, ^12^

### The patient chosen as the case study

This study was approved by the clinical ethics committee of the Affiliated Brain Hospital of Nanjing Medical University and participants in the LUAD cohort provided informed consent. The design and implementation of the study complied with the local regulations and the basic principles of the Declaration of Helsinki. The patient was staged according to the tumor, nodes, and metastases (TNM) staging system of the American Joint Committee on Cancer (7th version).

Quantitation of tumor epithelial cells and mesenchymal cells was studied by immunohistochemical and cell count methods. After obtaining consent from the patient, surgically obtained tissues and matching whole blood sample were used for testing with the YuanSuTM 450 tumor‐related gene panel. The testing was carried out by OrigiMed, a College of American Pathologists (CAP)‐accredited and Clinical Laboratory Improvement Amendments (CLIA)‐certified laboratory, in Shanghai, China. Somatic mutations were identified by comparing the patient's tumor DNA sample with matching whole blood sample.

### Gene‐set enrichment analysis

The gene sets of immune‐related pathway were retrieved from Kyoto Encyclopedia of Genes and Genomes (KEGG) database by R packages GAGE.^13^ ssGSEA algorithms^14^ implemented in R packages GSVA^15^ was used to estimate enrichment score of pathway.

### Statistical analysis

The Wilcoxon rank‐sum test was used to compare expression, TILs, and enrichment score. The Benjamini–Hochberg method was applied to correct the *p*‐value for multiple comparisons. The Spearman rank correlation test was performed to quantify the correlation of gene pairs. Kaplan–Meier survival curve was used to show the survival difference between the groups, and *p*‐value was estimated with log‐rank test. The hazard ratio (HR) and 95% confidence interval (CI) were estimated with Cox model. The multivariate Cox model was used to adjust potential confounding factors. All statistical analyses were performed with R software 3.5.3.

## RESULTS

### KEAP1 mutation is associated with lower infiltration of immune cells in LUAD

**FIGURE 1:**
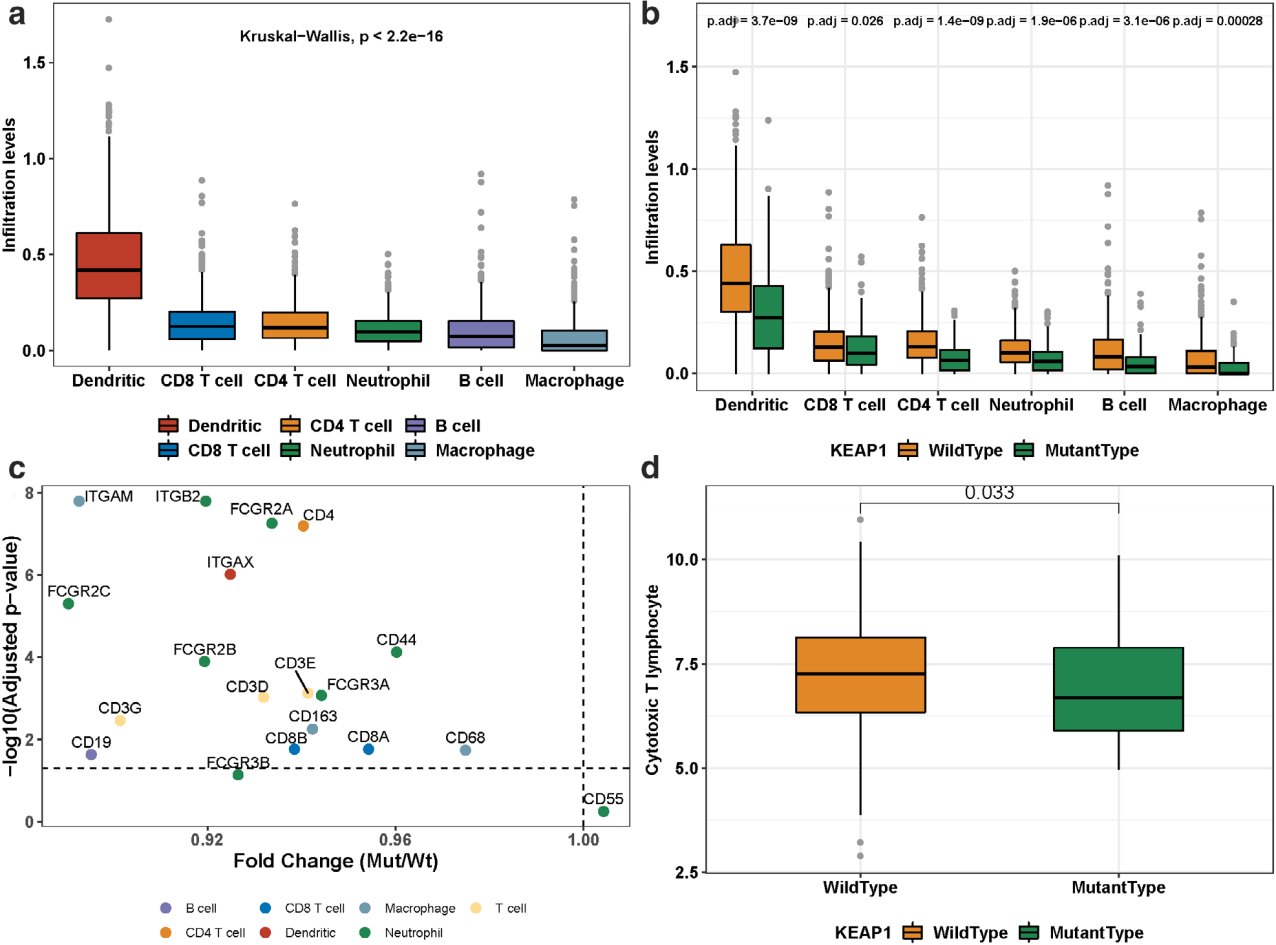
Tumor infiltrating leukocytes and KEAP1 mutation status. (a) Infiltration levels of six types of immune cells in LUADs. (b) Six types of immune cells and their infiltration levels in KEAP1‐mutated LUADs versus in KEAP1‐wild type LUADs. (c) Markers of the immune cells and their expressions in KEAP1‐mutated versus wild type LUADs. (d) Association of cytotoxic T lymphocytes (CTL) with KEAP1 mutation in LUADs. Wilcoxon rank‐sum test was used to compare difference, and *p*‐value was adjusted with Benjamini–Hochberg method

To evaluate the potential association between KEAP1 mutation status and immune microenvironment of the tumor in patients with LUAD, the abundance of infiltrating immune cells was estimated. We collected the abundance of six types of cells including B cells, CD4 T cells, CD8 T cells, dendritic, macrophages, and neutrophils, which were quantitatively calculated by gene expression profiles through RNA‐seq from TIMER database.^16^, ^17^ First, dendritic cells had the significantly highest infiltration level, followed by CD8 T cells, CD4 T cells, neutrophils, and B cells, whereas macrophage had the lowest infiltration level (Kruskal‐test, *p*‐value <2.2e−16) (Figure 1(a)). Second, the association between KEAP1 mutation status and the infiltration level of each cell type was studied. Among 543 LUAD patients, 96 patients were found to harbor KEAP1 mutation (frequency = 17.6%). Strikingly, the result showed that all of the six immune cell types had significantly lower infiltration levels in KEAP1‐mutated LUADs versus KEAP1‐wild type (Figure 1(b)), suggesting an overall low immune cell infiltration and low activity of immune environment within KEAP1‐mutated LUAD patients. In detail, significant differences of the infiltration between the KEAP1‐mutated and wild type LUADs were observed for dendritic cells (adjusted *p*‐value = 3.7e−9), CD8 T cells (adjusted *p*‐value = 0.026), CD4 T cells (adjusted *p*‐value = 1.4e−9), neutrophils (adjusted *p*‐value = 1.9e−6), B cells (adjusted *p*‐value = 3.1e−6), and macrophages (adjusted *p*‐value = 0.00028). Furthermore, we evaluated the abundance for 19 specific immune markers within the six immune cell types (Table S1) and found 17 markers significantly exhibited lower expression levels in KEAP1‐mutated LUADs than in KEAP1‐wild type LUADs (with adjusted *p*‐value <0.05) (Figure 1(c)), which was consistent with the result above. Only the marker of CD55 on neutrophil was found to have higher (but insignificantly) expression in KEAP1 mutated LUADs with fold change (FC) >1.0 and adjusted *p*‐value = 0.55. All of the results above confirmed that KEAP1‐mutated type LUADs are associated with the lower immune cell infiltration abundance. It is, therefore, assumable that KEAP1 mutated LUADs could possibly have lower tumor immunity, compared to the wild type patients. In addition, we estimated the cytotoxic T lymphocyte (CTL) level with the mean expression of its typical markers including CD8A, CD8B, GZMA, GZMB, and PRF1 by following Jiang et al.^18^ As shown in Figure 1(d), KEAP1‐mutated LUADs exhibited significantly lower CTL level than KEAP1‐wild type LUADs (*p*‐value = 0.033). This further suggested that KEAP1‐mutated LUAD patients may harbor the impaired tumor immunity.

### KEAP1 mutation is associated with lower human leukocyte antigen (HLA) expression in LUAD

**FIGURE 2:**
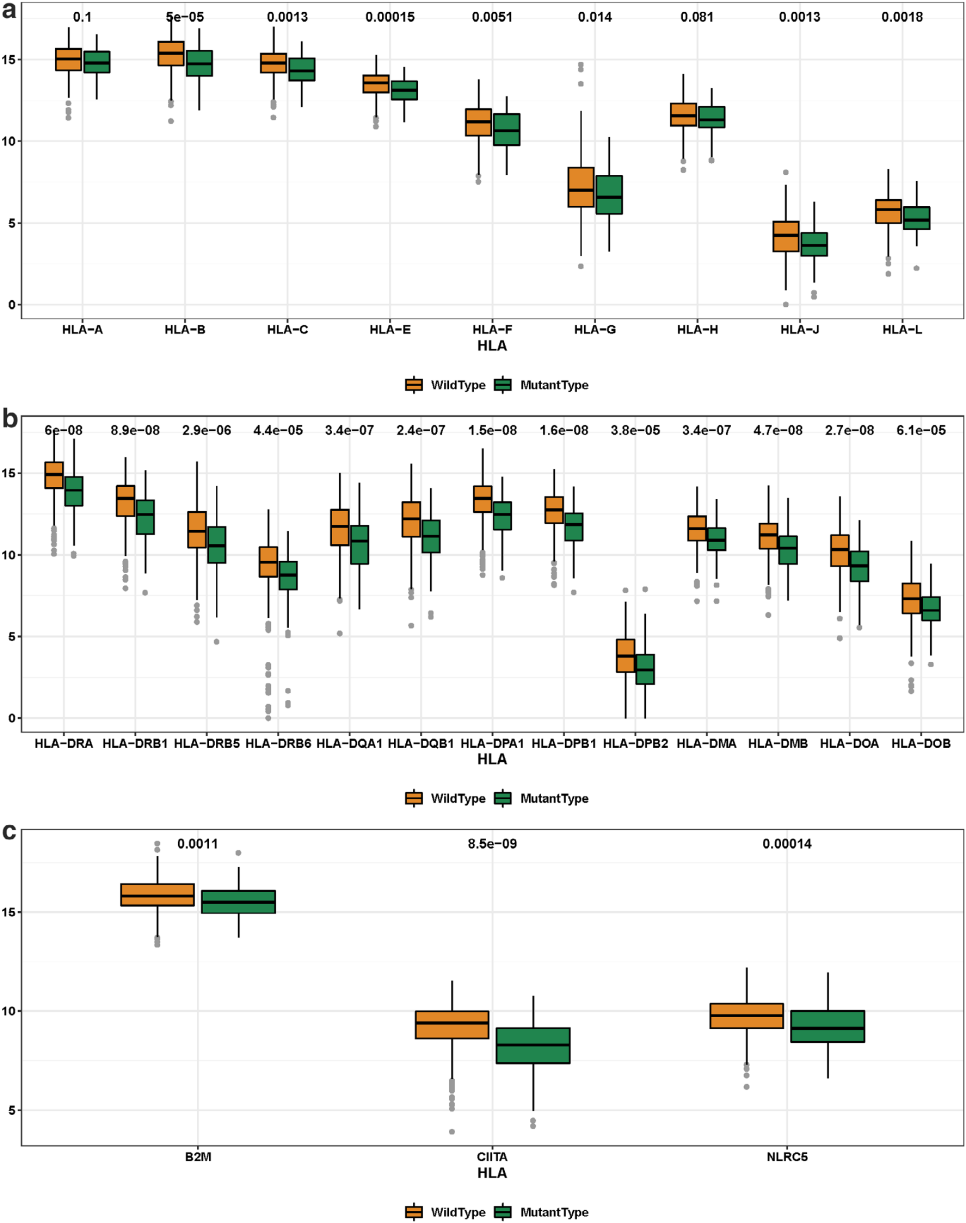
Association of KEAP1 mutation and HLA expression. (a) Boxplot shows expression of the type 1 HLA molecules in KEAP1‐wild type and KEAP1‐mutated LUADs. (b) Boxplot shows the expression of the type 2 HLA molecules in KEAP1‐wild type and KEAP1‐mutated LUADs. (c) Boxplot shows the expression of three important HLA related genes in KEAP1‐wild type and KEAP1‐mutated LUADs. The numeric above the boxplot indicates adjusted *p*‐value. Wilcoxon rank‐sum test was used to compare difference, and *p*‐value was adjusted with Benjamini–Hochberg method

In addition to immune infiltration cell, we were also interested in the expression of HLA molecules, in association with the mutation of KEAP1. As reported in IPD and IMGT/HLA database,^19^ 18 HLA class I and 13 HLA class II molecules have been identified in human genome. In 488 TCGA LUADs, we found that 9 molecules of class I and 13 ones of class II had expression values available. Among them, 7 HLA class I genes were found to have significantly lower expression level in KEAP1‐mutated LUADs than in KEAP1‐wild type LUADs (Figure 2(a)), and consistently, all of 13 HLA class II genes were found to have significantly lower expression level in KEAP1‐mutated LUADs (Figure 2(b)). We searched the literature and were interested in the expression of the HLA‐related genes like B2M, NLRC5, and CIITA, in association with KEAP1 mutation. As a result, B2M, as a component of HLA class I, exhibited significantly lower expression in KEAP1‐mutated LUADs (*p* = 0.001) (Figure 2(c)), and NLRC5 and CIITA, which play a major role in the transcription of HLA class I and II genes, showed the same trend, with the *p*‐values of 0.0014 and 8.5e−9, respectively (Figure 2(c)). Taken together, these findings indicated that KEAP1 mutation is likely associated with impaired presenting processes of tumor specific neoepitopes, therefore impairing tumor immunity.

### KEAP1 mutation is associated with lower activities of immune‐related pathway in LUAD

**FIGURE 3:**
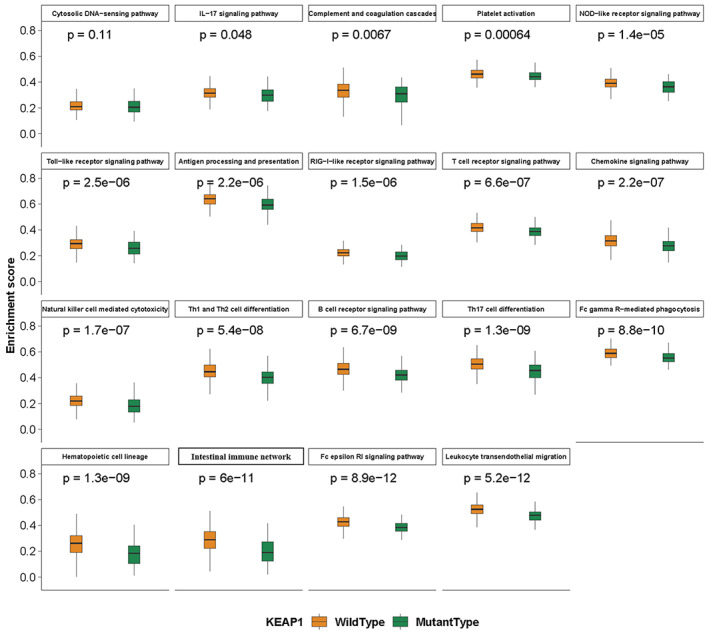
Association of KEAP1 mutation and immune‐related pathway activity. Boxplot shows the enrichment scores of the immune regulated pathway in KEAP1‐wild type and KEAP1‐mutated LUADs. Wilcoxon rank‐sum test was applied to calculate the *p*‐value

To further understand the effect of KEAP1 mutation on immune milieu, we performed enrichment analysis on 19 immune‐related pathways collected from the KEGG database, and the enrichment score for each pathway and each sample of 488 LUADs was calculated with the algorithm implemented in the Gene Set Variation Analysis (GSVA), a R software package^15^ to represent the activity of immune‐related pathway. As shown in Figure 3, all immune‐related pathways had lower enrichment scores in KEAP1‐mutated LUADs than in KEAP1‐wild type LUADs and 18 of them exhibited significantly lower enrichment scores with adjusted *p*‐value <0.05. Among them, both B cell receptor and T cell receptor signaling pathways were markedly downregulated with the significance of *p*‐values of 6.7e−9 and 6.6e−7, respectively. Meanwhile, the pathway involving natural killer cell mediated cytotoxicity was also significantly downregulated in KEAP1‐mutated LUADs (*p*‐value = 1.7e−7). Consistent with the HLA results above, antigen processing and presentation pathway was also affected (*p*‐value = 2.2e−6). Taken together, all of these results confirmed at the level of pathway that KEAP1 mutation could possibly have defective impact on tumor immunity.

### KEAP1 mutation is associated with chemokine network activity in LUAD

**FIGURE 4:**
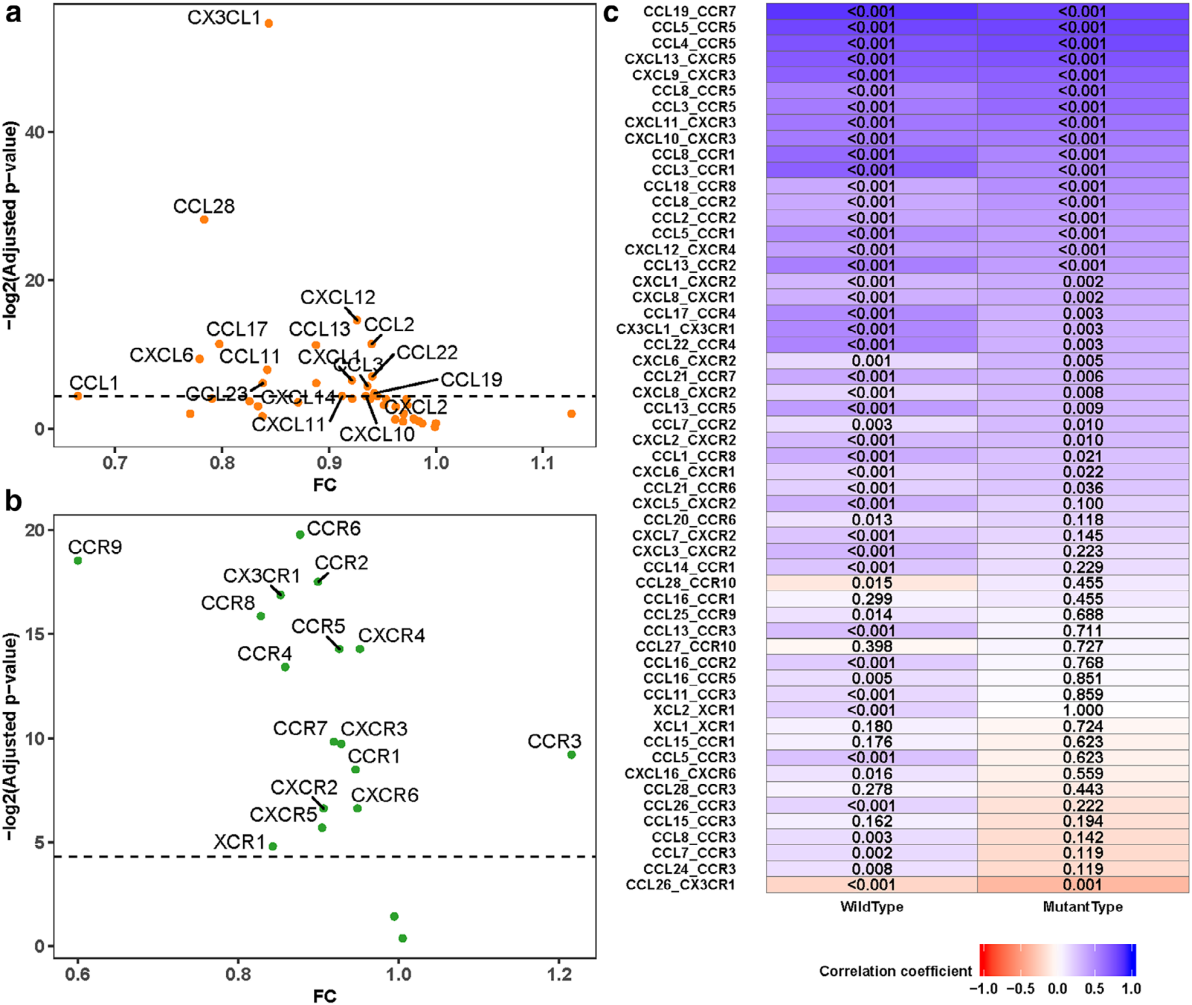
Association of KEAP1 mutation and chemokine network expression. Comparison of expression of the chemokines (a) and the chemokine receptors (b) for KEAP1‐mutated versus KEAP1‐wild type LUADs. Fold change (FC) was taken as x‐axis and negative log2 (adjusted *p*‐value) was taken as y‐axis. Wilcoxon rank‐sum test was used to compare difference, and *p*‐value was adjusted with Benjamini–Hochberg method. (c) The correlation coefficients of the pairs of the chemokine‐chemokine receptor in KEAP1‐wild type and KEAP1‐mutated LUADs, the numeric indicated the *p*‐value of coefficients

As we know, the migration and positioning of immune cells can be regulated by chemokines. Complex chemokine networks have been found in many cancers and they play important roles in the extent and phenotype of tumor lymphocyte infiltrate, as well as tumor cell growth, survival, migration, and angiogenesis.^20^, ^21^ Up to now, ~40 chemokines and 20 signaling chemokine receptors have been identified in human.^21^ Therefore, the correlation between KEAP1 mutated status and the expression levels of those genes was studies. Through the LUAD data set from TCGA, we found that 18 chemokines and 16 chemokine receptors had notably lower expression levels in KEAP1‐mutated samples (Figure 4(a),(b)). Interestingly, those genes are involved in regulating multiple immune‐related biological processes such as lymphocyte migration, neutrophil trafficking, T cell‐DC interactions, T helper cell response, etc.^21^

Moreover, we evaluated the interaction between chemokine and matched receptor. The results showed that many chemokines and chemokine receptor pairs had lower or even negative correlation coefficients (Figure 4(c)) in KEAP1‐mutated group compared to KEAP1‐wild type. Among those affected chemokine‐receptor pairs, some pairs such as CCL19, CCL21, and CCR7 were involved in T cell and dendritic homing to lymph node,^22^; whereas some pairs play a role in Th2 cells response^23^, ^24^ (e.g., CCL8 and CCR1, CCL13 and CCR2, CCL17 and CCR4), and some pairs contribute to^25^ T cell–dendritic interactions^26^ (e.g., CCL3 and CCR1 and CCL5 and CCR1). Given that chemokine network activity plays an important role in regulating the tumor immunity, it is assumable that the potential way of KEAP1 mutation impacting the immunity as suggested from the results above, might be likely related to damaging the chemokine network activity in LUADs.

### KEAP1 mutation predicts patient survival on immunotherapy

**FIGURE 5:**
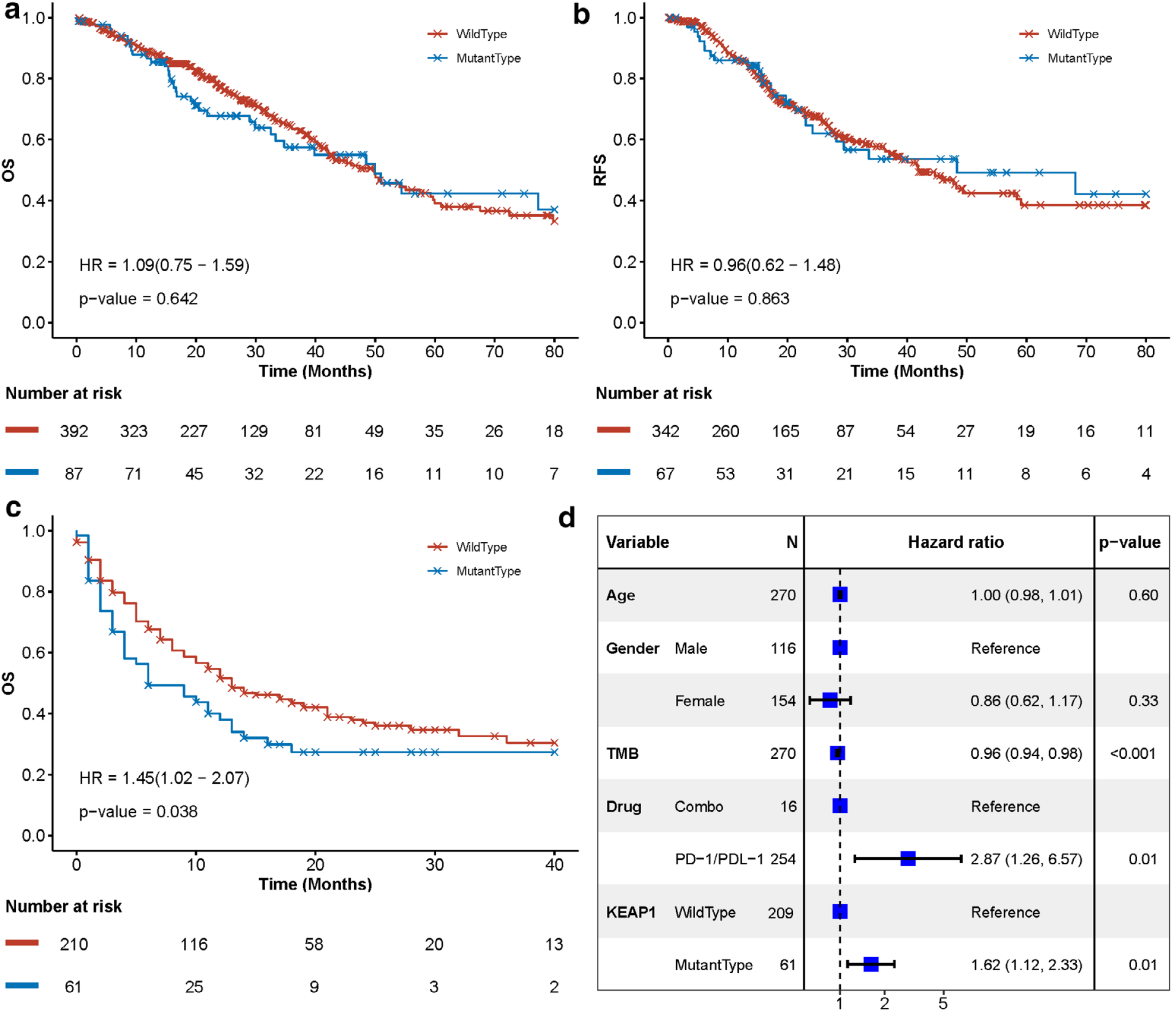
KEAP1 mutation is associated with survival after receiving immunotherapy in LUADs. Kaplan–Meier survival curves of KEAP1 mutated versus wild type LUADs for their overall survival (a) and release‐free survival (b). (c) Kaplan–Meier survival curves for the overall survival of KEAP1‐mutated versus wild type LUADs after receiving immunotherapy. (d) Multivariate Cox analysis with age, TMB, drug, and KEAP1 status as cofounding factors. Square data markers indicate estimated hazard ratios (HRs), and error bars represent 95% CIs. The *p*‐value was estimated with log‐rank test, and Cox analysis was applied to estimate HR

Finally, we investigated whether KEAP1 mutation can affect the survival of LUAD patients. In detail, neither overall survival (OS) nor relapse‐free survival (RFS) were found to have a significant correlation with KEAP1 status in LUADs (OS HR = 1.14, 95% CI = 0.8–1.64, log‐rank *p*‐value = 0.47; RFS HR = 1.09, 95% CI = 0.72–1.66, log‐rank *p* = 0.68) (Figure 5(a),(b)). However, our further studies suggest that KEAP1 mutation could be a predictive biomarker in immunotherapy with another dataset reported by Samstein et al.^11^ After immunotherapy (PD‐1 or PD‐L1 inhibitor), KEAP1‐wild type LUADs favored better survival than KEAP1‐mutated (OS HR = 1.45, 95% CI = 1.02–2.07, log‐rank *p*‐value = 0.038), with median survival 13 and 6 months, respectively. Multivariate Cox regression analysis was performed with various factors, including age, gender, TMB, immune drug‐type, and KEAP1 mutated status. KEAP1 was revealed to be an independent predictive biomarker for OS of LUAD patients (HR = 1.62, 95% CI = 1.12–2.33, *p*‐value = 0.01). This result suggests that KEAP1 mutated status could be identified as a predictive rather than a prognostic biomarker for survival on immunotherapy of LUAD patients.

### No response to immunotherapy for a LUAD patient harboring KEAP1 mutation despite high TMB

**FIGURE 6:**
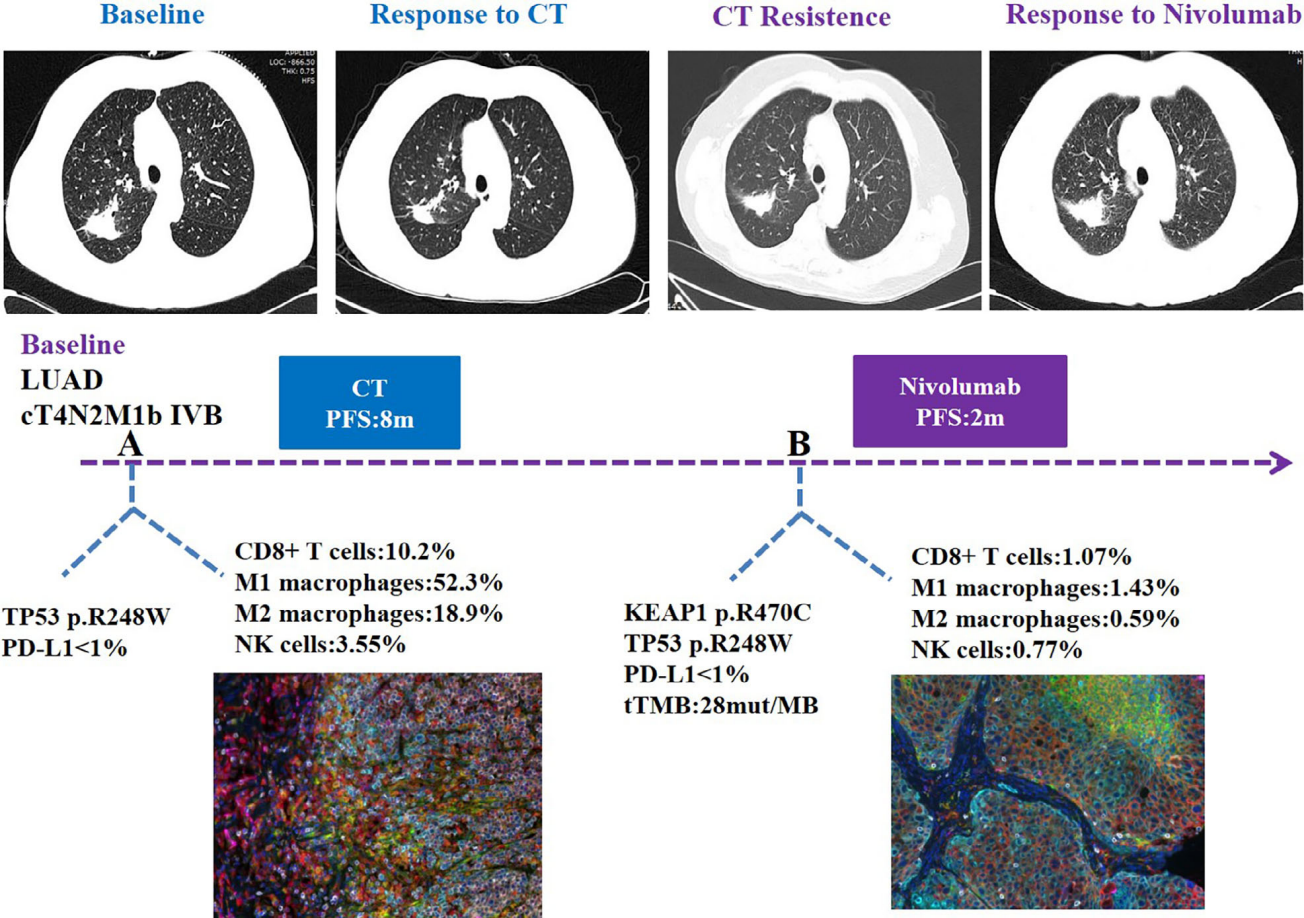
Representatives images and multiple immunocytochemistry of changes in a patient with mutated KEAP1 during treatment. Multiple immunocytochemistry revealed that the patient with mutated KEAP1 (p.R470C) owned low immune cell infiltration and low activity of immune environment. The patient showed no response to nivolumab as a second‐line treatment

A patient diagnosed with lung adenocarcinoma, whose chest computed tomography (CT) revealed a 3 cm × 4 cm mass in the right upper lobe with diffuse metastatic nodules in both lungs. The patient harboring TP53 p.R248W mutation had a favorable immune cell infiltration in tumor microenvironment. The chemotherapy regimen of “pemetrexed plus cisplatin” was administered as a first‐line treatment and had a response of partial response characterized by more than 8 months of control. A broad NGS test after recurrence was performed, showing KEAP1 p.R470C and TP53 p.R248W mutations along with a high TMB (28mut/MB). However, multiple immunocytochemistry revealed a low immune cell infiltration and low activity of immune environment. Unfortunately, the patient showed no response to a checkpoint inhibitor (nivolumab) as a second‐line treatment (Figure 6).

## DISCUSSION

In this study, we have performed a comprehensive investigation of KEAP1 gene mutation in LUAD patients with focus on its associations with immune microenvironment of tumor. Importantly, we have shown that LUADs with mutant type of KEAP1 gene carry a suppressed immune milieu from the following findings. First, the assessment of infiltration of immune cells has shown that the major immune cells including B cells, CD4 T cells, CD8 T cells, dendritic cells, macrophages, and neutrophils are downregulated within KEAP1 mutated LUADs and such correlation exhibits a highly statistical significance. Second, the key markers of immune cells are mostly downexpressed in KEAP1‐mutated LUADs, consistent with the first finding. Third, both class I and II of HLA molecules show significantly lower expression levels in KEAP1‐mutated LUADs. In addition, we have also comprehensively investigated the general immune‐related pathways and specific network of chemokine in this study. Overall, KEAP‐mutated LUADs compared to wild type ones have shown lower activities, and 17 pathways exhibit significantly lower pathway enrichment scores even after *p*‐value adjustment. Moreover, chemokine network, which is believed to regulate the migration and positioning of immune cells, has also been specifically studied in association with KEAP1 mutation in LUADs. Not only the multiple immune‐related biological processes, but also the interaction between chemokines and their receptors, have shown KEAP1 mutation could impair the immune pathways including the chemokine network, therefore explaining that LUADs with the wild type of this gene could potentially benefit from the immunotherapy. Last, these findings are in line with the previous study of Best et al.,^4^ in which the tumor immunosuppression environment was found to be associated with the loss of KEAP1 and PTEN in LUADs. Taken together, the results above highlight the possibility that KEAP1‐wild type tumors are associated with higher tumor immunity and, therefore, may be sensitive to immunotherapy, providing a new treatment modality and biomarker for those LUADs with KEAP1‐wild type.

Accordingly, we have evaluated the effects of KEAP1 mutation on disease prognosis from TCGA data and on immunotherapy using PD‐1/PD‐L1 blockers from previous published data,^11^ and the results have shown that there is no significant prognostic effect of KEAP1 mutation. On the contrary, it is therapeutically important to note that there is a predictive power of KEAP1 mutation on the immunotherapy: KEAP1‐wild type LUADs favored better survival after immunotherapy than KEAP1‐mutated LUADs. The latter result has verified that significant differences of immunogenomic were found between mutated KEAP1 and wild type groups in terms of core immune signatures.^27^ Therefore, we could conclude that the immune environment is significantly changed within the KEAP1‐mutated LUADs and this change has clinical implications, especially for the immunotherapy using PD‐L1 blockers.

Our study has limitations. We only have data available for KEAP1 status, survival data after immunotherapy, and a separate set of data for KEAP1 status and RNA‐seq data for defining immune milieu. Unfortunately, we do not have samples for assessing all of the three entities simultaneously. Therefore, all the conclusions above have been made based on correlation approach. LUAD patients with all the three measurements available in a future study will further validate the findings in this study. This study demonstrates the potential effect of KEAP1 mutation on tumor immune milieu of LUAD subtype of NSCLC patients, highlighting the potential efficacies of the immunotherapy treatment using PD‐L1 blockers in KEAP1‐wild type LUADs.

## CONFLICT OF INTEREST

None of the authors reported a conflict of interest related to the study.

## Supporting information

**Table S1** Marks for each type of immune cell
