# Supplementary material for: Lung adenocarcinoma patients with KEAP1 mutation harboring low immune cell infiltration and low activity of immune environment
Source: Thorac Cancer. 2021 Jul 30;12(18):2458–67. doi: 10.1111/1759-7714.14089 (PMC8447911; doi:10.1111/1759-7714.14089)
Supplement: Supplementary file 1 — Table S1 Marks for each type of immune cell [file TCA-12-2458-s001.docx]

| Table S1. Marks for each type of immune cell | | |  |
| --- | --- | --- | --- |
| Cell type | Protein | GeneSymbol | Expression |
| B cell | CD19 | CD19 | Positive |
| T cell | CD3 | CD3D | Positive |
| T cell | CD3 | CD3E | Positive |
| T cell | CD3 | CD3G | Positive |
| CD4 T cell | CD4 | CD4 | Positive |
| CD8 T cell | CD8 | CD8A | Positive |
| CD8 T cell | CD8 | CD8B | Positive |
| Dendritic | ITGAX | ITGAX | Positive |
| Macrophage | ITGAM | ITGAM | Positive |
| Macrophage | CD68 | CD68 | Positive |
| Macrophage | CD163 | CD163 | Positive |
| Neutrophil | CD16 | FCGR3A | Positive |
| Neutrophil | CD16 | FCGR3B | Positive |
| Neutrophil | CD18 | ITGB2 | Positive |
| Neutrophil | CD32 | FCGR2A | Positive |
| Neutrophil | CD32 | FCGR2B | Positive |
| Neutrophil | CD32 | FCGR2C | Positive |
| Neutrophil | CD44 | CD44 | Positive |
| Neutrophil | CD55 | CD55 | Positive |
